# Health Services Impact of Hospital-Older Adult Center Partnerships for People Living with Dementia

**DOI:** 10.1093/geroni/igaf122.727

**Published:** 2025-12-31

**Authors:** Divya Bhagianadh, Clara Scher, Emily Greenfield, Ceara Somerville, Caitlin Coyle, Ayse Akincigil

**Affiliations:** University of Arkansas, Fayetteville, Arkansas, United States; Weill Cornell Medicine, New York, New York, United States; Rutgers University, New Brunswick, New Jersey, United States; University of Massachusetts Boston, Boston, Massachusetts, United States; University of Massachusetts Boston, Boston, Massachusetts, United States; Rutgers University, New Brunswick, New Jersey, United States

## Abstract

There is growing enthusiasm for policies and practices that promote cross-sectoral collaboration to improve care for people aging in community with dementia. Our study aimed to expand empirical evidence in this area by examining the impact of partnerships between hospitals and older adult centers on healthcare utilization and costs among community-dwelling older adults with dementia. Older adult centers are community-based organizations that provide essential services and social connections for participants. Our study uses a novel, statewide hierarchical dataset of older adults with dementia in Massachusetts nested within the geographic catchment areas of municipally-run older adult centers that did or did not report partnerships with hospitals. Outcomes including number of hospital stays, readmissions and total Medicare payments were from 2018 - 2019 Medicare claims data. We employed a multilevel mixed-effects generalized linear model. Results suggested that individuals with dementia living in communities where older adult centers engaged in partnerships with hospitals had significantly fewer hospital stays. Among subgroups of dementia patients with multiple comorbidities and among dual-eligible older adults, these partnerships were associated with significantly lower levels of healthcare utilization and Medicare costs. In addition, people with dementia at the intersection of other minoritized identities (e.g., racialized as African American, lower socioeconomic status) were less likely to live in communities reporting hospital-older adult center partnerships. Our findings highlight the importance of continued research, policy, and practice on the interfaces across health and social care settings to improve outcomes for people with dementia, especially for those aging in place with greater precarity.

